# Effects of n-3 Polyunsaturated Fatty Acid-Enriched Hen Egg Consumption on the Inflammatory Biomarkers and Microvascular Function in Patients with Acute and Chronic Coronary Syndrome—A Randomized Study

**DOI:** 10.3390/biology10080774

**Published:** 2021-08-14

**Authors:** Željka Breškić Ćurić, Ana Marija Masle, Aleksandar Kibel, Kristina Selthofer-Relatić, Ana Stupin, Zrinka Mihaljević, Ivana Jukić, Marko Stupin, Anita Matić, Nataša Kozina, Petar Šušnjara, Brankica Juranić, Nikolina Kolobarić, Vatroslav Šerić, Ines Drenjančević

**Affiliations:** 1 Scientific Center of Excellence for Personalized Health Care, Josip Juraj Strossmayer University of Osijek, HR-31000 Osijek, Croatia; zeljka.breskic@gmail.com (Ž.B.Ć.); anamarija.masle@kbco.hr (A.M.M.); alekibel@mefos.hr (A.K.); ksrelatic@mefos.hr (K.S.-R.); zrinka.mihaljevic@mefos.hr (Z.M.); ivana.jukic@mefos.hr (I.J.); mstupin@mefos.hr (M.S.); anita.matic@mefos.hr (A.M.); natasa.kozina@mefos.hr (N.K.); psusnjara@mefos.hr (P.Š.); bjuranic@fdmz.hr (B.J.); nbdujmusic@mefos.hr (N.K.); 2 Department of Internal Medicine, General Hospital Vinkovci, HR-32100 Vinkovci, Croatia; 3 Department of Rheumatology, Clinical Immunology and Allergology, Osijek University Hospital, HR-31000 Osijek, Croatia; 4 Department of Physiology and Immunology, Faculty of Medicine, Josip Juraj Strossmayer University of Osijek, HR-31000 Osijek, Croatia; 5 Department for Cardiovascular Disease, Osijek University Hospital, HR-31000 Osijek, Croatia; 6 Department of Internal Medicine, Faculty of Medicine, Josip Juraj Strossmayer University of Osijek, HR-31000 Osijek, Croatia; 7 Department of Pathophysiology, Physiology and Immunology, Faculty of Dental Medicine and Health, Josip Juraj Strossmayer University of Osijek, HR-31000 Osijek, Croatia; 8 Department of Nursing and Palliative Medicine, Faculty of Dental Medicine and Health, Josip Juraj Strossmayer University of Osijek, HR-31000 Osijek, Croatia; 9 Department of Clinical Laboratory Diagnostics, Osijek University Hospital, HR-31000 Osijek, Croatia; seric.vatroslav@kbo.hr

**Keywords:** n-3 polyunsaturated fatty acids, functional food, coronary artery disease, inflammation, oxidative stress, microcirculation

## Abstract

**Simple Summary:**

There is a strong potential of n-3 polyunsaturated fatty acid (n-3 PUFA) consumption to reduce cardiovascular risk and prevent adverse outcomes in existing cardiovascular diseases. This study aimed to test the effect of n-3 PUFA supplementation in the form of enriched hen eggs on serum lipid and free fatty acid profiles, inflammatory and oxidative stress biomarkers, and microvascular reactivity in patients with acute and chronic coronary artery disease. Consumption of three n-3 PUFA-enriched hen eggs for three weeks had a favorable effect on serum free fatty acid profile (a lower n-6/n-3 PUFA ratio) and mild anti-inflammatory effects but did not significantly affect microvascular reactivity in patients with coronary artery disease. Because consumption of both regular and n-3 PUFA eggs had no negative effects on any of the measured biological and functional vascular parameters, the results of the present study indicate that eggs can be safely consumed in the daily diet of patients with coronary artery disease.

**Abstract:**

This study aimed to test the effect of n-3 polyunsaturated fatty acid (PUFA)-enriched hen egg consumption on serum lipid and free fatty acid profiles, inflammatory and oxidative stress biomarkers, and microvascular reactivity in patients with coronary artery disease (CAD). Forty CAD patients participated in this study. Of those, 20 patients had acute CAD (Ac-CAD), and 20 patients had chronic CAD (Ch-CAD). The control group (N = 20) consumed three regular hen eggs/daily (249 mg n-3 PUFAs/day), and the n-3 PUFAs group (N = 20) consumed three n-3 PUFA-enriched hen eggs/daily (1053 g n-3 PUFAs/day) for 3 weeks. Serum n-3 PUFA concentration significantly increased (in all CAD patients), while LDL cholesterol and IL-6 (in Ac-CAD patients), and hsCRP and IL-1a (in all CAD patients) significantly decreased in the n-3 PUFAs group. Glutathione peroxidase (GPx) activity significantly decreased, and forearm skin microvascular reactivity in response to vascular occlusion (postocclusive reactive hyperemia (PORH)) remained unchanged in both the n-3 PUFAs and control groups in total CAD, Ac-CAD, and Ch-CAD patients. Potentially, n-3 PUFA-enriched hen eggs can change the free fatty acid profile to a more favorable lower n6/n3 ratio, and to exhibit mild anti-inflammatory effects but not to affect microvascular reactivity in CAD patients.

## 1. Introduction

Coronary artery disease (CAD), characterized by atherosclerotic plaque accumulation in the epicardial arteries, is the most common type of heart disease and the leading cause of death in developed countries in both men and women [1,2]. Chronic CAD patients have stable disease with or without a history of earlier acute coronary syndrome (ASC), while patients with acute CAD have complete or partial coronary artery obstruction due to plaque rupture or thrombus formation [1,2]. CAD is often accompanied by established metabolic syndrome with insulin-resistant hyperglycemia, dyslipidemia, abdominal obesity, and hypertension, all of which contribute to the etiopathogenesis of CAD and also to its complications, worsening the outcome of the disease [3,4]. Moreover, both CAD and metabolic syndrome are clinically manifested with established endothelial dysfunction (ED), which presents one of the earliest symptoms underlying various cardiovascular diseases (CVDs) (e.g., hypertension, diabetes mellitus, obesity, hyperlipidemia, and atherosclerosis) [5,6]. ED is characterized by proinflammatory and prothrombotic phenotypes, attenuated vasodilation responses, increased endothelial permeability, platelet aggregation, and leukocyte adhesion, resulting in impaired endothelium-dependent vasodilation, systemic endothelial activation, and endothelial–leukocyte interaction [6,7]. Increased oxidative stress levels [8], as well as generation of high levels of proinflammatory cytokines (e.g., IL-1, IL-6, and TNFα), resulting in vascular or systemic inflammation [9], are key pathophysiological mechanisms mediating the development of ED [10]. For example, hypercholesterolemia and particularly high levels of low-density lipoprotein (LDL) cholesterol have been shown to increase oxidative stress and vascular inflammation, which both lead to the reduced bioavailability of NO [10]. Oxidative stress affects the production of vasoactive and proinflammatory prostaglandins, leading to impaired endothelium-dependent vasoreactivity [11].

There is a strong potential of n-3 polyunsaturated fatty acid (n-3 PUFA) consumption, especially eicosapentaenoic acid (EPA) and docosahexaenoic acid (DHA), to reduce blood triglyceride levels, arterial blood pressure, thrombosis incidence, and the risk of sudden death due to myocardial infarction and prevent cardiac arrhythmias [12,13,14]. Consumption of foods rich in n-3 PUFAs (fatty fish and vegetables, seeds and nuts rich in n-3 PUFAs), as well as pharmacological foods rich in n-3 PUFAs containing fish oil, krill oil, cod liver oil, algae oil, or n-3 PUFAs-enriched foodstuff (called functional foods), are known to increase antioxidant capacity and thus enhance vascular reactivity in both patients with CAD and healthy individuals [15]. Moreover, consumption of approximately 1 g of PUFAs per day has been recommended in CVDs patients due to their positive cardiovascular effects, especially because there are no significant interactions between n-3 PUFA supplements and the number of drugs these patients often take [16]. 

n-3 PUFAs (as well as n-6 PUFAs, such as arachidonic acid) are precursors of oxylipins. Their subgroups of eicosanoids include prostaglandins (PGs), thromboxanes (TXs), and leukotrienes (LTs), which are the key mediators and regulators of inflammation [17,18], as well as endothelium-dependent vascular responses and function [19]. Moreover, oxylipins act as signaling molecules in an autocrine or paracrine way and achieve their effect by binding to receptors activated by peroxisome proliferators (PPARs) [20]. Interestingly, significantly more oxylipins are released by the coronary arteries than by all classes of the aorta and pulmonary arteries [21]. Thus, it could be hypothesized that increased dietary intake of n-3 PUFAs may change the oxylipins’ profile in the coronary arteries and have a beneficial effect on vascular function and low-grade inflammation in patients with CAD.

Our recently published work showed that consumption of two n-3 PUFA-enriched hen eggs per day for three weeks (~410 mg of n-3 PUFAs per day) had immunomodulatory effects, without affecting the reactivity of peripheral microcirculation in healthy young subjects [22]. However, an increased amount of n-3 PUFAs in the daily diet to 1053 g of n-3 PUFAs/day for 3 weeks increased serum free n-3 PUFA concentration and enhanced skin microvascular reactivity while reducing proinflammatory and increasing anti-inflammatory cytokine serum concentration in healthy young subjects [23]. Furthermore, patients with metabolic syndrome who consumed two boiled n-3 PUFAs-enriched eggs per day for 5 weeks had significantly reduced total cholesterol, LDL cholesterol, and blood pressure levels and increased HDL cholesterol levels [24]. Altogether, this supports the rationale to investigate the potentially beneficial effect of n-3 PUFAs intake in the form of functional foods on vascular endothelium-dependent reactivity and other relevant markers of CV health in patients with CAD. 

Thus, the aim of the present study was to (a) determine the effect of consumption of n-3 PUFAs-enriched hen eggs on serum lipid and free fatty acid profiles, pro- and anti-inflammatory cytokines, and biomarkers of oxidative stress and (b) to examine its potential effect on forearm skin microvascular reactivity in patients with existing acute and chronic CAD.

## 2. Materials and Methods

### 2.1. Study Design and Study Population

This study was designed as a randomized, double-blind, placebo-controlled interventional experiment. It was conducted at the Laboratory for Clinical and Sport Physiology, Department of Physiology and Immunology at the Faculty of Medicine Osijek, University of Osijek, Osijek, Croatia. Recruitment started on 1 September, 2019, and subject follow up finished on 31 December, 2019. Forty patients with existing CAD of both sexes (9 women and 31 men) participated in the study. Patients were recruited at the Department for Cardiovascular Disease, Osijek University Hospital, Osijek, Croatia. Eligibility criteria for the study included: (1) patients with acute coronary syndrome: (a) with ST-segment elevation (STEMI); (b) without ST elevation (NSTEMI); (c) unstable angina pectoris; and (2) patients with chronic coronary syndrome (diagnosed according to ESC guidelines for the diagnosis and management of chronic coronary syndromes, 2019). Exclusion criteria for the study included: malignant disease, hereditary metabolic and systemic diseases, autoimmune diseases, active treatment with monoclonal antibodies, immunosuppressive drugs, systemic corticosteroids, recent surgery (3 months), recent severe trauma (6 months), condition after resuscitation (3 months), severe renal impairment, liver failure, active bleeding, recent stroke (6 weeks), neurodegenerative diseases, epilepsy, significant anemia (Hg M < 110; W < 100), chronic respiratory failure and hypoxemia, sepsis, active tuberculosis, untreated thyroid disease, and active abuse of alcohol and drugs. Subjects did not use functional foods enriched in n-3 PUFAs or n-3 PUFA supplements (e.g., capsules) before entering the study protocol. The drug therapy taken by CAD patients during the present study is described in Table 1. All Ch-CAD patients used statins and other standard drug therapy for CAD (ACE inhibitors, beta blockers, antiplatelet drugs), and some of them used ezetimibe depending on lipid profile. In Ac-CAD patients, high-dose statin therapy was started during hospitalization, along with other cardiac therapy. Written informed consent was obtained from each subject. The study protocol and procedures conformed with the standards set by the latest revision of the Declaration of Helsinki and were approved by the Ethical Committee of Osijek University Hospital (Approval Number: R2:19326-7/2016). The present study is part of a registered clinical trial on the effect of n-3 PUFA-enriched food on microvascular reactivity at (ClinicalTrials.gov access date 7 April 2021) (ID: NCT02720250, Omega-3 Fatty-Acid-Enriched Food and Microvascular Reactivity).

### 2.2. Study Protocol

During the study, subjects had two study visits: the first on the first day and the second on the day immediately after the end of the diet protocol. The dates of arrival of the subjects were scheduled in advance by the researcher. Study visits were organized in the morning, and subjects were instructed not to eat or take caffeine before the visit and not to perform any strenuous activity 24 h before the visit. Data collection took place at the Laboratory for Clinical and Sport Physiology, Department of Physiology and Immunology at the Faculty of Medicine, Josip Juraj Strossmayer University of Osijek, Osijek, Croatia.

Study intervention involved eating three hard-boiled hen eggs per day for three weeks (21 days, total of 63 eggs). Subjects eating n-3 PUFA-enriched hen eggs (1053 mg of n-3 PUFAs per day) comprised the experimental n-3 PUFAs group (20 subjects), while those eating regular hen eggs (249 mg of n-3 PUFAs per day) comprised the control group (20 subjects). All hen eggs (regular and n-3 PUFAs enriched) were produced on the same farm and were the same M commercial size. Recruited patients were individually assigned to the diet protocol. The simple randomization procedure was performed using a coin (letter-1 or head-2) by the unbiased associate who also administered the appropriate eggs to each subject (according to randomization). Both subjects and researchers did not know to which group the subjects belonged until the end of the 3-week dietary protocol (eggs were labeled as numbers 1 or 2 before being distributed to the laboratory). 

Established procedures by the research group from the Faculty of Agrobiotechnical Sciences Osijek, Josip Juraj Strossmayer University of Osijek, Croatia, were used to produce n-3 PUFA-enriched hen eggs, which involved the replacement of soybean oil (5%) with a mixture of fish (1.5%) and linseed (3.5%) oil in the feeding mixture to each laying hen. Each n-3 PUFA egg contained on average 351 mg of n-3 PUFAs (ALA 230.5 mg/egg, EPA 15.1 mg/egg, DHA 105.5 mg/egg), and each control egg contained on average 83 mg of n-3 PUFAs (ALA = 36 mg/egg, EPA = 0 mg/egg, DHA = 47 mg/egg). 

Subjects were instructed to boil the eggs for approximately 10 min before consumption and to eat them at the same time of day (breakfast). All subjects were instructed not to change their usual diet during the study other than egg intake, to eat only the 63 eggs provided by the study protocol, and not to eat any other food rich in n-3 PUFAs or n-3 PUFAs supplementation during the study protocol. To ensure maximum compliance to the diet protocol, patients were frequently contacted by telephone and instructed to keep to their specific diet diary. 

The primary outcome of the study included a change in microvascular reactivity in response to vascular occlusion after the dietary protocol. Secondary outcomes included change in serum protein concentration of pro- and anti-inflammatory cytokines and chemokines, change in serum free fatty acid profile and lipid profile, as well as change in biomarkers of oxidative stress level, including thiobarbituric acid-reactive substances (TBARS), ferric-reducing ability of plasma (FRAP), and enzyme activity of glutathione peroxidase (GPx) following the respective dietary protocol.

### 2.3. Anthropometric and Hemodynamic Measurements

Subject height (m), weight (kg), waist circumference (cm), and hip circumference (cm) were measured to calculate body mass index (BMI) and waist-to-hip ratio (WHR) Procedures for body composition and body fluid status measurement are described in Supplementary Material. An automated oscillometric sphygmomanometer (OMRON M3, OMRON Healthcare Inc., Osaka, Japan) was used for blood pressure (BP) and heart rate (HR) measurements. BP and HR were measured in a seated position three consecutive times. The final BP and HR values were the mean of these three repeated measurements.

### 2.4. Serum Free Fatty Acid Profile, Lipid Profile, and Biochemical Markers Analysis

Venous blood samples, taken at both study visits, were analyzed for full blood count, von Willebrandt factor, urea, creatinine, urates, plasma electrolytes (sodium, potassium, calcium), iron, transferrin, fasting blood glucose, high-sensitivity C-reactive protein (hsCRP), and fasting lipid profile (total cholesterol, high-density lipoprotein (HDL) cholesterol, low-density lipoprotein (LDL) cholesterol, and triglycerides) at the Department of Clinical Laboratory Diagnostics, Osijek University Hospital, Osijek, Croatia. 

Gas chromatography–tandem mass spectrometry (GC–MS/MS) (Thermo Fisher GC Trace 1300 coupled with a TSQ 9000 Triple Quadrupole) was used for serum free fatty acid profile analysis, according to the established protocols described in a recent paper of our study group [23]. These analyses were performed at the BIOCentre’s Bioanalytical Laboratory, BIOCentre—incubation center for biosciences, Zagreb, Croatia.

### 2.5. Assay of Protein Concentration of Pro- and Anti-Inflammatory Cytokines and Chemokines in Serum

Serum concentrations of interleukin 1a (IL-1a), interleukin (IL-6), tumor necrosis factor alpha (TNF-α), interleukin 10 (IL-10), and the chemokine monocyte chemoattractant protein-1 (MCP-1) were measured with Invitrogen ProcartaPlex antibody-based magnetic bead reagent kits and panels for multiplex protein quantitation using the Luminex 200 instrument platform. Quantitation was performed using ProcartaPlex Analyst free software and expressed as concentration in picograms per milliliter. Measurements were performed in the Laboratory for Immunology and Allergology Diagnostics, Osijek University Hospital, Osijek, Croatia. 

### 2.6. Measurement of Thiobarbituric Acid-Reactive Substances (TBARS) and Ferric-Reducing Ability of Plasma (FRAP)

The thiobarbituric acid-reactive substances (TBARS) method uses spectrophotometry to measure products of lipid peroxidation and presents a measure of oxidative stress level. Because other substances bind to thiobarbituric acid (including proteins), the method is nonspecific, and trichloroacetic acid was added to the sample to precipitate the proteins. The ferric-reducing ability of plasma (FRAP) method also uses spectrophotometry to measure the antioxidant capacity of blood samples. Fe^3+^-TPTZ (2,4,6-tris(2-pyridyl)-s-triazine) is reduced to Fe^2+^-TPTZ in the presence of antioxidants, and a blue discoloration occurs. The general procedures for TBARS and FRAP measurements were performed according to a previously described protocol in our laboratory [25].

### 2.7. Spectrophotometric Antioxidant Enzyme Activities Assay 

Enzyme activity assay of glutathione peroxidase (GPx) in serum samples was performed using a Lambda 25 UV–vis spectrophotometer equipped with the UV WinLab 6.0 software package (PerkinElmer, Waltham, MA, USA) according to the established protocol in the Biochemistry Laboratory at the Department of Biology, University of Osijek [25,26]. Measured activities of GPx were expressed as units of the enzymes per milligram of protein (U/mg protein). The concentration of proteins in serum samples (mg/mL) was determined following the manufacturer’s protocols for Bradford reagent (Bradford Reagent B6916, Sigma Aldrich, Saint Louis, MO, USA) at 595 nm using bovine serum albumin as a standard.

### 2.8. Assessment of Microvascular Reactivity in Response to Vascular Occlusion 

Postocclusive reactive hyperemia (PORH) was determined by laser Doppler flowmetry (MoorVMS-LDF, Axminster, UK) to assess change in overall microvascular reactivity. LDF measurement was performed in a temperature-controlled environment and started after 30 min resting in a supine position to acclimatize. The laser Doppler probe was attached to the volar forearm skin, 13–15 cm from the wrist, at the same place at each study visit. After a 5 min baseline measurement, the PORH test was performed by occlusion of the brachial artery using a pneumatic cuff, which was inflated on the upper arm to 30–50 mmHg above the systolic BP for 1 min. Blood flow in skin microcirculation was expressed in arbitrary perfusion units and determined by software calculating the area under the curve (AUC) during baseline flow, occlusion, and reperfusion (1 min period). The final result was the difference between the percentage of flow change during reperfusion and occlusion in relation to baseline (R–O% increase). The protocol for PORH LDF measurement is described in earlier papers of our research group [27,28]. 

### 2.9. Statistical Analysis

Results are reported as the arithmetic mean ± standard deviation (SD) for normally distributed data and as the median and interquartile range for non-normally distributed data. The sample size required to show a potentially significant effect was calculated based on preliminary data collected from 8 subjects. To detect differences in primary outcome reported in this study (LDF PORH) with a level of significance of 0.05 and a statistical power of 80% for paired *t*-test, the needed sample size was 15 subjects per group. The normality of data distribution was assessed by the Shapiro–Wilk normality test. To test differences between measurements performed before and after the respective diet protocol within groups, the paired *t*-test for normally distributed data was used. When variables were non-normally distributed, the Wilcoxon rank-sum test was used. Differences in baseline measurements between the control and n-3 PUFAs groups were tested with the Student’s *t*-test for parametric and the Mann–Whitney test for nonparametric distributions. Between-group differences in the after-intervention measurement were tested with analysis of covariance (ANCOVA) with the baseline value (before) as the covariate. *p* < 0.05 was considered statistically significant. Statistical analysis was performed by SigmaPlot, version 11.2 (Systat Software, Inc., Chicago, IL, USA).

## 3. Results

**Figure 1:**
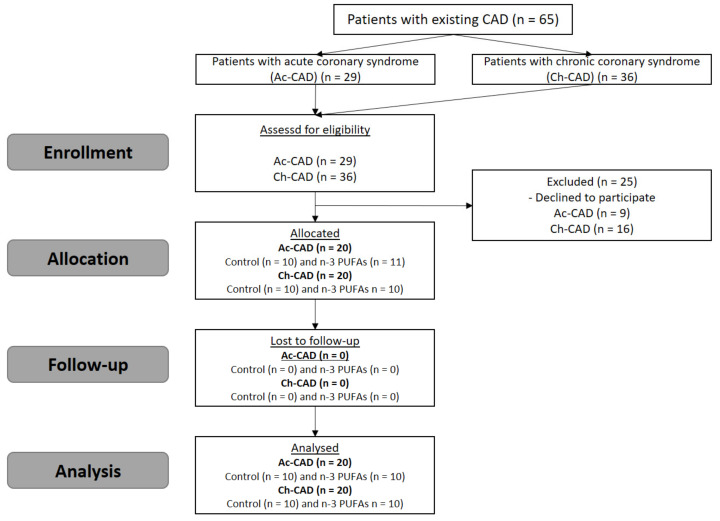
Flowchart of participants through each stage of the study. CAD—coronary artery disease; Ac-CAD—acute coronary artery disease; Ch-CAD—chronic coronary artery disease.

The flow of participants through each stage of the study is described in Figure 1. A total of forty participants completed the three-week dietary protocol and consumed three eggs per day. Initial clinical characteristics of the study population are presented in Table 2 and Table 3. All CAD patients also had metabolic syndrome, defined according to the International Diabetes Federation (IDF) consensus criteria [29]. Generally, participants were overweight to obese, indicated by a BMI above 30 kg/m^2^ and WHR <0.90, as well as hyperglycemic with a fasting glucose level above 6.4 mmol/L. Patients had a systolic and diastolic BP level within the recommended values, as expected in the CVD patient population due to antihypertensive therapy (e.g., ACEi/ARB, beta blocker, nitrates; Table 1). Furthermore, patient hsCRP values were above the referent range in both Ac-CAD and Ch-CAD patients at the moment entering the study protocol. Initially, all subjects had normal full blood counts, renal function, serum electrolytes, and fasting total cholesterol level but increased triglycerides and LDL cholesterol levels. 

### 3.1. Anthropometric and Hemodynamic Parameters

There was no significant difference in BMI, WHR, systolic BP, diastolic BP, mean arterial pressure (MAP), and HR values following consumption of n-3 PUFA-enriched hen eggs compared to the initial measurements within the n-3 PUFAs in all CAD populations. In the control group, only HR level significantly decreased following consumption of regular eggs compared to baseline Results of body composition and body fluid status measurement in study population are described in Supplementary Material Table S1. There was no statistically significant difference in the BMI, WHR, systolic BP, diastolic BP, MAP, and HR between the control and n-3 PUFAs groups following the corresponding dietary protocols (adjusted for baseline). All the values were similar within the n-3 PUFAs or control group in both the Ac-CAD and Ch-CAD subgroups of patients after the corresponding dietary protocol (Table 2).

### 3.2. Biochemical Parameters

Total study population (CAD). Consumption of n-3 PUFA-enriched eggs significantly decreased erythrocytes, hemoglobin, and hematocrit values within the n-3 PUFAs group following the respective dietary protocol, while values of other measured biochemical parameters remained unchanged. Creatinine and urate levels significantly decreased, while there was no other significant difference in measured biochemical parameters after consumption of regular hen eggs compared to the initial measurements within the controls. Urea, creatinine, and hsCRP levels were significantly higher (albeit within the population reference range), while calcium and transferrin levels were significantly lower in the n-3 PUFAs group compared to the control group following the respective diet protocol (adjusted for baseline values) (Table 3). Laboratory reference range for measured biochemical parameters is listed in Supplementary Material (Table S2).

Ac-CAD subgroup. There was no significant difference in measured biochemical parameters after consumption of n-3 PUFA-enriched hen eggs compared to the initial measurements within the n-3 PUFAs group. Consumption of regular hen eggs significantly decreased serum creatinine and urate levels following regular egg consumption compared to the initial measurement in the control group. Calcium level was significantly lower, while urea, creatinine, and hsCRP levels were significantly higher in the n-3 PUFAs group compared to the control group following the respective diet protocol (adjusted for baseline values) (Table 3).

Ch-CAD subgroup. Consumption of n-3 PUFA-enriched eggs significantly decreased only erythrocytes, hemoglobin, and hematocrit values within the n-3 PUFAs group following the respective dietary protocol. Consumption of regular hen eggs significantly decreased serum creatinine level, while there was no other significant difference in biochemical parameters following regular egg consumption compared to the initial measurement in the control group. Creatinine level was significantly higher in n-3 PUFAs compared to the control group (within the reference range) following the respective diet protocol, while there was no significant difference in other biochemical parameters between the control and n-3 PUFAs groups after the corresponding dietary protocols (adjusted for baseline values) (Table 3).

### 3.3. Serum Lipid Profile

Total study population (CAD). Consumption of n-3 PUFA-enriched eggs significantly decreased total cholesterol and LDL cholesterol levels in the n-3 PUFAs group following the respective dietary protocol, while triglycerides and HDL cholesterol levels remained unchanged. Consumption of regular hen eggs significantly decreased the serum triglyceride levels, while there was no significant difference in other serum lipids (total cholesterol, LDL and HDL cholesterol) following regular egg consumption compared to the initial measurement in the control group. Serum lipid profile did not significantly differ between the control and n-3 PUFAs groups after the corresponding dietary protocols (adjusted for baseline).

Ac-CAD subgroup. Consumption of n-3 PUFA-enriched eggs significantly decreased total cholesterol and LDL cholesterol levels, while triglycerides and HDL cholesterol levels remained unchanged within the n-3 PUFA group compared to baseline measurements. Consumption of regular hen eggs significantly decreased total cholesterol and triglycerides levels, while there was no significant difference in HDL and LDL cholesterol levels following regular egg consumption compared to the initial measurement in the control group. There was no significant difference in serum lipid profile between the control and n-3 PUFAs groups after the corresponding dietary protocols (adjusted for baseline).

Ch-CAD subgroup. Serum lipid values did not significantly differ following n-3 PUFAs or regular hen egg consumption compared to the initial measurements within the n-3 PUFAs or control group or between these groups. All these results are presented in Table 4.

### 3.4. Serum Free Fatty Acid Profile

A total of 37 free fatty acids in the serum sample were analyzed, and only the values of fatty acids of which concentrations were detected (above the limit of quantification) are presented in Table 5. Serum concentration of EPA significantly increased, and concentrations of 16:0 palmitic acid, C16:1[cis-9] palmitoleic acid, C18:1[cis-9] oleic acid, C20:3[cis-8,11,14] dihomo-gamma-linolenic acid, and C20:4[cis-5,8,11,14] arachidonic acid significantly decreased after n-3 PUFA-enriched egg consumption compared to the initial measurement within the n-3 PUFAs group. Serum concentrations of other measured free fatty acids were similar before and after the respective dietary protocol within the n-3 PUFAs group. In total, consumption of n-3 PUFA-enriched eggs decreased the serum n-6/n-3 ratio by approximately 34%. Serum concentrations of measured free fatty acids remained unchanged, and the n-6/n-3 ratio even increased by approximately 8% following regular egg consumption compared to baseline measurements within the control group. Serum C18:1[cis-9] oleic acid concentration was significantly lower in the n-3 PUFA group than in the control group following completion of the corresponding dietary protocol (adjusted for baseline values) (Table 5). Still, these results should be interpreted with caution because serum free fatty acid profile was analyzed in only four CAD patients from each study group (control and n-3 PUFAs).

### 3.5. Serum Pro- and Anti-Inflammatory Cytokines and Chemokines Protein Concentration

Total study population (CAD). Only IL-1a serum concentration significantly decreased after n-3 PUFAs egg consumption compared to baseline measurements. On the contrary, only serum concentration of IL-1a significantly increased after consumption of regular hen eggs compared to baseline. There was no significant difference in measured cytokines and chemokines between the control and n-3 PUFAs groups after the corresponding dietary protocols (adjusted for baseline values) (Table 6).

Ac-CAD subgroup. Consumption of n-3 PUFA-enriched eggs significantly decreased serum IL-6 concentration, while IL-1a, TNF-α, IL-10, and MCP-1 concentrations remained unchanged within the n-3 PUFA group compared to baseline measurements. There was no significant difference in measured cytokine and chemokine levels after consumption of regular hen eggs compared to the initial measurements within the control group. The concentrations of cytokines and chemokines did not significantly differ between the control and n-3 PUFAs groups after the corresponding dietary protocols (Table 6).

Ch-CAD subgroup. None of the measured pro- and anti-inflammatory biomarkers (IL-1a, TNF-α, IL-6, IL-10, MCP-1) were significantly altered by the dietary interventions, irrespectively of the dietary protocol. Serum cytokines and chemokines concentrations did not significantly differ between the control and n-3 PUFAs groups after the corresponding dietary protocols (Table 6).

### 3.6. Biomarkers of Oxidative Stress and Antioxidative Defense

Total study population (CAD) and Ac-CAD and Ch-CAD subgroups. Serum GPx activity significantly decreased, and TBARS and FRAP remained unchanged within the n-3 PUFA group compared to baseline measurements in the total population (CAD) and in the Ac-CAD and Ch-CAD subgroups. Consumption of regular hen eggs significantly decreased GPx and did not change TBARS and FRAP compared to baseline in controls in the total population (CAD) population. FRAP and serum GPx activity decreased, and TBARS remained unchanged following regular egg consumption compared to the initial measurement in the Ac-CAD subgroup’s controls, while there was no significant difference in TBARS, FRAP, and serum GPx activity after consumption of regular hen eggs compared to the initial measurements within the Ch-CAD subgroup’s controls. There was no significant difference in TBARS, FRAP, and GPx serum activity between the control and n-3 PUFAs groups after the corresponding dietary protocols in the total population (CAD) population and Ac-CAD and Ch-CAD subgroups (adjusted for baseline) (Table 7).

### 3.7. Postocclusive Reactive Hyperemia (PORH) of Forearm Skin Microcirculation

**Figure 2:**
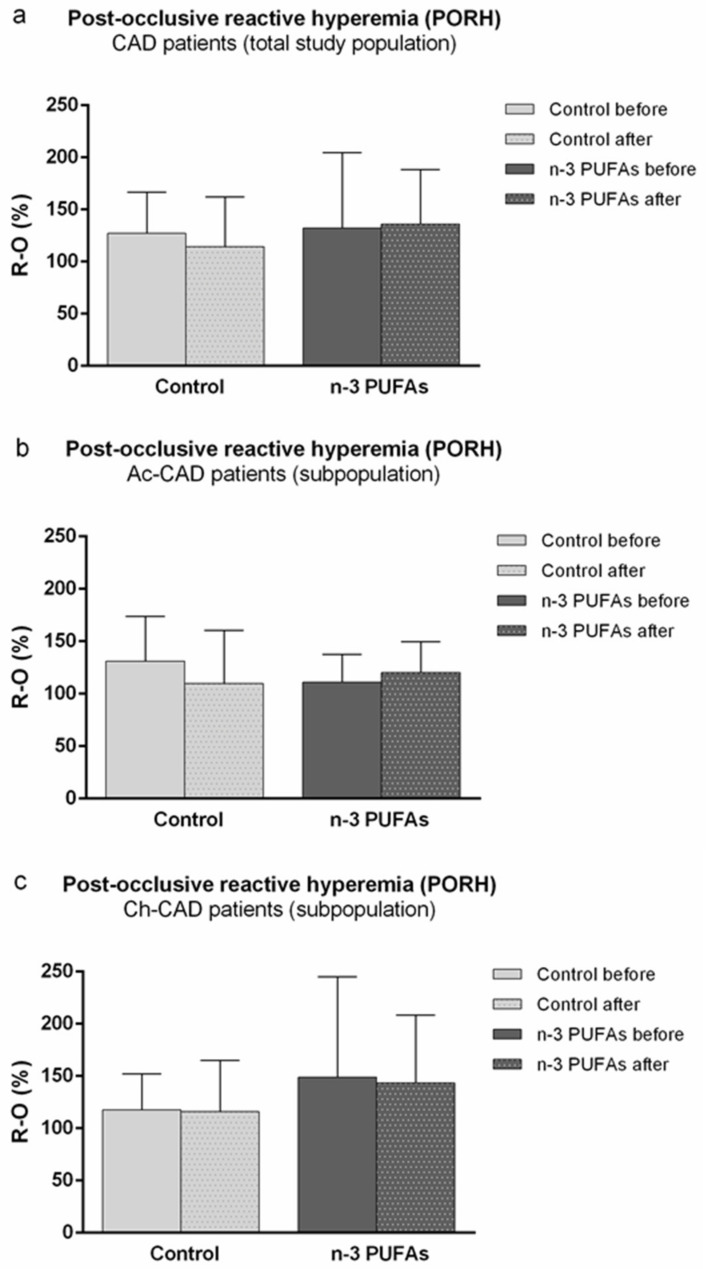
Three-week regular (control group) and n-3 PUFA (n-3 PUFAs group)-enriched hen egg consumption effect on skin microvascular response to vascular occlusion (postocclusive reactive hyperemia (PORH)) in (**a**) CAD patients (total study population), (**b**) Ac-CAD patients, and (**c**) Ch-CAD patients. PORH is expressed as the difference between the percentage of flow change during reperfusion and occlusion in relation to baseline (R-O%). Data are presented as the arithmetic mean ± standard deviation (SD). n-3 PUFA—n-3 polyunsaturated fatty acids, CAD—coronary artery disease; Ac-CAD—acute CAD patients; Ch-CAD—chronic CAD patients.

N-3 PUFA-enriched hen egg consumption did not induce significant changes in PORH compared to baseline measurements, neither when data were analyzed for the total study population (CAD patients) (Figure 2a), nor when values were compared within the Ac-CAD (Figure 2b) and Ch-CAD subgroups (Figure 2c), respectively. PORH was similar between initial measurement and measurement following regular hen egg consumption in the total study population (CAD patients) (Figure 2a), as well as in the Ac-CAD (Figure 2b) and Ch-CAD (Figure 2c) subgroups of patients.

## 4. Discussion

This was the first interventional experimental study that investigated the effects of n-3 PUFA-enriched functional food intake on serum free fatty acid and lipid profile, markers of inflammation and oxidative stress level, and microcirculatory function in CAD patients, including those with stable disease (Ch-CAD) and those with ACS (Ac-CAD). The main findings of the present study are as follows: (a) consumption of n-3 PUFA-enriched eggs significantly reduced the n-6/n-3 PUFA ratio in CAD patients, with an observed significant reduction of LDL cholesterol levels in Ac-CAD patients and in the total study population (CAD) (with no such observed effects in the Ch-CAD subgroup); (b) n-3 PUFA-enriched hen eggs significantly decreased hsCRP and proinflammatory cytokine IL-1a levels in the total study population (CAD) and decreased proinflammatory cytokine IL-6 level in the Ac-CAD subgroup; and finally (c) n-3 PUFA-enriched hen eggs did not induce significant changes in microvascular function of CAD patients (both Ch-CAD and Ac-CAD). Taken together, consumption of three n-3 PUFA-enriched eggs per day (1053 mg PUFAs/day) for three weeks had mild anti-inflammatory effects and induced a more favorable serum free fatty acid profile but did not significantly alter microvascular function in patients with existing CAD. 

### 4.1. n-3 PUFA-Enriched Hen Eggs and Serum Lipid Profile in CAD Patients

n-3 PUFA supplementation (EPA and DHA) has the potential to reduce serum lipids (especially triglycerides) in hyperlipidemic individuals [30,31] and improve endothelial function and arterial stiffness in hypertensive patients with hypertriglyceridemia and high cardiovascular risk [32]. Still, the doses necessary to obtain observed effects are high; i.e., taking >4 g/day of EPA- and/or DHA-enriched food sources reduced serum triglycerides (by 9–26%) in normolipidemic to borderline hyperlipidemic but otherwise healthy individuals [33]. A recent clinical trial of our research group demonstrated that consumption of 1053 g n-3 PUFAs/day for 3 weeks in enriched hen eggs did not induce significant changes in serum lipid profile compared to baseline measurements in healthy individuals (e.g., triglyceride levels decreased by approximately 8%, *p* > 0.05) [23]. The present study showed that n-3 PUFA-enriched hen eggs significantly decreased total and LDL cholesterol in all CAD patients. This is attributed to a significant decrease in total and LDL cholesterol levels following n-3 PUFA consumption in Ac-CAD patients only. However, in the Ac-CAD subgroup, total cholesterol and triglyceride levels significantly decreased, similar to patients consuming regular hen eggs (controls). It is important to emphasize that the levels of total cholesterol, triglycerides, and LDL cholesterol in all CAD patients were within the reference values, especially in the subpopulation of Ch-CAD patients, most likely because they were taking statins prior and during the enrollment in the present study. Furthermore, statins were de novo initiated in patients with ACS (Ac-CAD). Thus, it is plausible that the lowering of total and LDL cholesterol levels in Ac-CAD patients in both n-3 PUFAs and controls could be attributed to statin therapy rather than to the specific dietary protocol they were subjected to. Therefore, these results indicate that consumption of n-3 PUFAs did not induce a significant additional reduction in total cholesterol, triglyceride, and LDL cholesterol levels in Ch-CAD patients. However, an important finding of the present study is that consumption of hen eggs had no negative effect on lipid profile and turned out to be completely safe in the population of CAD patients. 

### 4.2. n-3 PUFA-Enriched Hen Eggs and Serum Pro- and Anti-Inflammatory Cytokines and Chemokines Protein Concentration in CAD Patients

IL-1 is a known immunomodulatory cytokine that increases the production of other proinflammatory chemokines and cytokines (specifically IL-6) and thus starts a cascade promoting atherosclerosis and plaque destabilization in CV patients, characterized by increased endothelial expression of cell adhesion molecules, endothelial and smooth muscle cell proliferation, and increased vascular permeability and macrophage activation [34]. IL-6 is associated with an increased risk of future myocardial infarction (MI) in healthy middle-aged men [35] and could be clinically used as a predictor for the development of ischemic heart disease and other cardiovascular endpoints (PRIME study [36]). Interestingly, n-3 PUFAs supplementation in the form of capsules (2g n-3 PUFAs per day/3 months) significantly decreased IL-6 and TNFα levels in patients with chronic heart failure [37], and CRP and IL-6 levels were significantly decreased in overweight women with an inflammatory phenotype [38]. Consistent with these results, the present study demonstrated that n-3 PUFA-enriched hen egg consumption significantly decreased IL-1 (in all CAD patients) and IL-6 levels (only in Ac-CAD patients), while TNF-α, IL-10, and MCP-1 levels remained unchanged following the respective diet protocol. Clearly, a decrease in the proinflammatory cytokines IL-1 and IL-6 (such as in the Ac-CAD subgroup) following a diet rich in n-3 PUFA-enriched eggs may have direct implications for coronary artery disease pathogenesis and recovery from an acute incident. However, it was previously shown that atorvastatin treatment compared to diet recommendation alone resulted in significant reductions in LDL cholesterol, TNFα, IL-1, IL-6, and CRP in hypercholesterolemic patients [39]. Therefore, any benefit of a diet, including functional foods intake (e.g., n-3 PUFA-enriched hen eggs used in the present study), is not meant as a substitute for pharmacological therapy but to provide an additional beneficial effect on cardiovascular health.

### 4.3. n-3 PUFA-Enriched Hen Eggs and Oxidative Stress in CAD Patients

The existing evidence suggests that oxidative stress may play a crucial role in cardiac and vascular abnormalities related to different types of CVDs [40]. n-3 PUFA supplementation significantly increases serum total antioxidant capacity and glutathione peroxidase (GPx) activity and decreases malondialdehyde (MDA) compared to placebo [41]. In addition, a Mediterranean type of diet that is rich in n-3 PUFAs is associated with lower levels of oxidative stress biomarkers and is widely recommended not only to cardiovascular patients but to the general population as well [42]. Interestingly, low or medium doses of n-3 PUFA intake in the form of enriched hen eggs increase superoxide dismutase (SOD) levels in young and healthy individuals, indicating that n-3 PUFAs could be involved in the physiological processes associated with oxidative balance in healthy individuals [22]. The present results demonstrated that intake of n-3 PUFA-enriched hen eggs significantly decreased GPx activity but did not affect TBARS (a measure of oxidative stress) and FRAP (a measure of antioxidant capacity) levels in patients with CAD. Such a decrease in GPx plasma activity due to n-3 PUFA intake could be potentially attributed to a lower oxidative stress level with pharmacological therapy and thus to a lower stimulus for GPx activation, but, for now, we can only speculate about the mechanisms mediating the relationship between n-3 PUFAs and antioxidant enzymes activity in patients with diagnosed CAD.

### 4.4. n-3 PUFA-Enriched Hen Eggs and Skin Microvascular Reactivity in CAD Patients

Skin microvascular PORH is widely used for the assessment of microvascular function [43]. Skin microvascular function is impaired in many CVDs and thus could be considered as a representative vascular bed for investigating microvascular function in various CVDs [44]. n-3 PUFAs may reduce the risk of CVDs, at least in part, by improving vascular function. For example, microvascular endothelium-dependent function was improved by n-3 PUFA (EPA+DHE, EPA, or DHE alone) supplement intake for longer periods (6, 12, and 17 weeks) in patients with hyperlipidemia, overweight patients, and patients with diabetes mellitus type 2 [32,45,46,47,48,49]. Despite great heterogeneity in the study design (participants’ age and sex; dose, form, and duration of n-3 PUFA supplementation; different CV risk factors), these trials indicate that supplementation of n-3 PUFAs (especially EPA+DHE) has the potential to improve endothelial dysfunction in microcirculation in individuals with increased cardiovascular risk. The results of the present, controlled, randomized study demonstrated that consumption of 1053 mg of PUFAs per day in the form of enriched hen eggs for three weeks did not induce significant changes in PORH of skin microcirculation in patients with existing CAD, irrespectively of the duration of CAD. These results could potentially be attributed to an insufficiently high dose or, compared to other studies, a relatively short period of PUFA supplementation in the present study, which is possibly insufficient to induce functional vascular effects in altered microcirculation in patients with existing CAD. Therefore, further clinical studies and better standardization of the n-3 PUFA supplementation protocol (dose, duration, form of supplementation) are needed to achieve clearer results on the effect of consumption of n-3 PUFAs, especially in the form of functional foods, in patients with CVDs. 

*Limitation of the study:* Serum free fatty acid profile was determined in only four CAD patients from each study group (control and n-3 PUFAs). The diet of participants was not monitored throughout the study. Although the sample size of patients is generally sufficient for statistical analysis, considering the multicausality of coronary artery syndrome and complexity of the disease, the results need to be taken and interpreted cautiously. In the present study, therapy administered by the attending physician is deemed “optimal” and reflects the best available individual medical care. Our study evaluated the enriched diet as an additional “layer” of intervention controlled with regular (placebo) eggs. In this respect, the possible influence of concomitant medication on the results of the egg diet was reduced to a minimum, as far as is reasonable and ethically acceptable. The evaluation of the concomitant effects of pharmacological therapy taken by patients may be considered as a limitation of the study.

## 5. Conclusions

The present study showed the anti-inflammatory potential of n-3 PUFAs egg consumption in CAD patients. Potentially, n-3 PUFA-enriched hen eggs can change the free fatty acid profile to a more favorable lower n6/n3 ratio, and decrease total cholesterol and LDL cholesterol levels in an Ac-CAD subpopulation of patients. n-3 PUFA-enriched egg consumption did not induce a significant effect on the oxidative stress level (TBARS) and antioxidant capacity (FRAP) in CAD patients but decreased GPx activity, which suggests that the oxidative stress level was low. Finally, this study clearly demonstrated that consumption of both regular and n-3 PUFA eggs had no negative effects on any of the measured biological and functional vascular parameters in CAD patients, which indicates that eggs can be safely consumed in the daily diet of CAD patients. Due to the limited number of CAD patients involved in the present study, a future larger study is necessary to confirm the observed effects.

## Figures and Tables

**Table 1:** Existing drug treatment used in the study population: patients with existing coronary artery disease (CAD).

Type of Drug	Acute CAD Patients (Ac-CAD)	Chronic CAD Patients (Ch-CAD)
	Number of Patients Taking the Therapy	
Acetylsalicylic acid	20	16
Beta blocker	14	18
ACEi or ARB	19	19
Statins	20	20
Fenofibrate	0	2
Ezetimibe	0	4
Nitrate	2	3
Clopidogrel or ticagrelor	20	1
Trimetazidine	6	6
Warfarin or NOAC	0	4

ACEi—angiotensin-converting enzyme inhibitor; ARB—angiotensin receptor blocker; NOAC—novel oral anticoagulants.

**Table 2:** The effect of regular (control group) and n-3 PUFA-enriched hen eggs (n-3 PUFAs group) consumption on anthropometric and hemodynamic parameters in the total study population (CAD) and in the two subgroups of patients (Ac-CAD patients and Ch-CAD patients).

Parameter	Control			n-3 PUFAs			Between-Group Effect (After) Adjusted for Baseline
	Before	After	*p* Value *^a^*	Before	After	*p* Value *^a^*	*p* Value *^b^*
**Total study population (CAD)**							
BMI (kg/m^2^)	30.5 ± 3.3	30.5 ± 3.1	0.699	31.1 ± 6.5	31.2 ± 6.6	0.736	0.057
WHR	0.94 ± 0.12	0.94 ± 0.12	0.283	0.94 ± 0.08	0.94 ± 0.12	0.175	0.363
SBP (mmHg)	119 ± 13	126 ± 14	0.054	125 ± 16	132 ± 16	0.080	0.840
DBP (mmHg)	79 ± 11	83 ± 8	0.168	83 ± 12	86 ± 12	0.541	0.801
MAP (mmHg)	93 ± 11	97 ± 8	0.090	97 ± 13	101 ± 13	0.180	0.631
HR (bpm)	77 ± 14	71 ± 15	0.010	65 ± 11 *^c^*	68 ± 8	0.157	0.088
**Acute CAD patients (Ac-CAD)**							
BMI (kg/m^2^)	31.2 ± 2.7	31.4 ± 2.6	0.347	31.8 ± 6.9	31.9 ± 7.3	0.503	0.122
WHR	0.93 ± 0.15	0.93 ± 0.15	0.363	0.97 ± 0.08	0.96 ± 0.10	0.378	0.057
SBP (mmHg)	118 ± 10	129 ± 15	0.115	127 ± 17	137 ± 14	0.063	0.541
DBP (mmHg)	83 ± 10	87 ± 7	0.296	83 ± 12	88 ± 12	0.331	0.407
MAP (mmHg)	95 ± 9	101 ± 9	0.188	98 ± 13	104 ± 12	0.187	0.279
HR (bpm)	79 ± 11	73 ± 13	0.054	71 ± 11	72 ± 7	0.657	0.054
**Chronic CAD patients (Ch-CAD)**							
BMI (kg/m^2^)	29.7 ± 3.7	29.7 ± 3.4	0.809	30.5 ± 6.4	30.4 ± 6.2	0.633	0.163
WHR	0.95 ± 0.10	0.94 ± 0.09	0.619	0.90 ± 0.06	0.89 ± 0.05	0.327	0.276
SBP (mmHg)	118 ± 15	124 ± 12	0.241	127 ± 11	128 ± 16	0.859	0.657
DBP (mmHg)	76 ± 11	78 ± 7	0.242	86 ± 7 *^c^*	84 ± 12	0.429	0.051
MAP (mmHg)	90 ± 12	94 ± 6	0.230	100 ± 7 ^c^	99 ± 13	0.744	0.067
HR (bpm)	75 ± 17	70 ± 17	0.132	61 ± 8 *^c^*	64 ± 8	0.185	0.643

Data are presented as the mean ± standard deviation (SD) (normally distributed data) or as the median and interquartile range (non-normally distributed data). Total study population: control N = 20 (women N = 3, men N = 17) and n-3 PUFAs N = 20 (women N = 6, men N = 14). Acute CV patients (Ac-CV): control N = 10 (women N = 1, men N = 9) and n-3 PUFAs N = 10 (women N = 1, men N = 9). Chronic CAD patients (Ch-CAD): control N = 10 (women N = 2, men N = 8) and n-3 PUFAs N = 10 (women N = 5, men N = 5). CAD—coronary artery disease; BMI—body mass index; WHR—waist-to-hip ratio; SBP—systolic blood pressure; DBP—diastolic blood pressure; MAP—mean arterial pressure; HR—heart rate; BPM—beats per minute. *^a^* Analysis of difference between before and after within the group (paired *t*-test or Wilcoxon signed-rank test). *^b^* Analysis of covariance (ANCOVA) model included the baseline value as the covariate. *^c^* *p* < 0.05 difference in baseline value between Control and n-3 PUFAs (Student’s *t*-test).

**Table 3:** The effect of regular (control group) and n-3 PUFA-enriched hen eggs (n-3 PUFAs group) consumption on biochemical parameters in the total population (CAD) and in the two subgroups (Ac-CAD patients and Ch-CAD patients).

Parameter	Control			n-3 PUFAs			Between-Group Effect (After) Adjusted for Baseline
	Before	After	*p* Value *^a^*	Before	After	*p* Value *^a^*	*p* Value *^b^*
**Total study population (CAD)**							
Erythrocytes (×10^−12^/L)	4.9 ± 0.3	4.8 ± 0.5	0.987	4.8 ± 0.7	4.6 ± 0.6	0.020	0.190
Hemoglobin (g/L)	150 ± 12	149 ± 14	0.659	146 ± 11	140 ± 11	0.008	0.114
Hematocrit (%)	42.6 ± 3.5	42.3 ± 4.3	0.677	41.8 ± 3.1	40.1 ± 2.9	0.011	0.083
Leukocytes (×10^−9^/L)	9.3 ± 2.9	8.7 ± 2.1	0.136	8.8 ± 2.4	8.3 ± 2.0	0.156	0.870
Thrombocytes (×10^−9^/L)	263 ± 65	243 ± 56	0.055	240 ± 49	221 ± 44	0.084	0.321
vWf	1.4 ± 0.5	1.3 ± 0.6	0.229	1.6 ± 0.6	1.5 ± 0.7	0.317	0.343
Urea (mmol/L)	6.8 ± 1.9	6.7 ± 2.5	0.866	7.3 ± 3.7	7.2 ± 4.4	0.708	0.030
Creatinine (µmol/L)	80 [69–92]	76 [65–83]	0.002	92 ± 56	89 ± 59	0.102	0.001
Urates (µmol/L)	374 ± 112	331 ± 95	0.005	379 [295–434]	379 [288–425]	0.165	0.797
Sodium (mmol/L)	138 ± 3	139 ± 3	0.340	139 ± 2	138 ± 2.2	0.086	0.180
Potassium (mmol/L)	4.4 ± 0.3	4.5 ± 0.4	0.605	4.3 ± 0.3	4.2 ± 0.3	0.218	0.623
Calcium (mmol/L)	2.48 [2.40–2.58]	2.46 [2.37–2.53]	0.225	2.47 [2.35–2.54]	2.42 [2.38–2.49]	0.105	0.002
Iron (µmol/L)	14.9 ± 4.6	13.3 ± 3.5	0.161	13.6 ± 4.1	14.5 ± 3.1	0.457	0.131
Transferrin (g/L)	2.49 [2.41–2.80]	2.57 [2.35–2.75]	0.832	2.38 ± 0.38	2.30 ± 0.35	0.081	0.024
Glucose (mmol/L)	6.4 [5.6–8.6]	6.1 [5.6–7.9]	0.245	6.5 [5.6–7.8]	6.0 [5.5–7.7]	0.712	0.851
hsCRP (mg/L)	3.8 [1.1–8.0]	1.8 [0.8–6.9]	0.622	2.1 [1.1–18.6]	2.4 [0.9–3.7]	0.096	<0.001
**Acute CAD patients (Ac-CAD)**							
Erythrocytes (×10^−12^/L)	4.9 ± 0.3	5.1 ± 0.5	0.148	5.0 ± 0.9	4.9 ± 0.8	0.215	0.320
Hemoglobin (g/L)	154 ± 9	155 ± 11	0.478	150 ± 7	145 ± 8	0.132	0.121
Hematocrit (%)	43.4 ± 3.7	44.1 ± 4.2	0.412	43.0 ± 2.8	41.1 ± 2.3	0.124	0.072
Leukocytes (×10^−9^/L)	9.6 [8.3–11.8]	8.4 [7.3–10.1]	0.074	9.5 ± 2.5	9.2 ± 1.9	0.632	0.505
Thrombocytes (×10^−9^/L)	268 ± 81	241 ± 60	0.118	253 ± 59	230 ± 52	0.299	0.467
vWf	1.6 ± 0.6	1.4 ± 0.7	0.064	1.6 ± 0.5	1.5 ± 0.8	0.798	0.896
Urea (mmol/L)	6.8 ± 2.2	6.0 ± 1.1	0.261	7.3 ± 2.7	6.5 ± 2.7	0.228	0.038
Creatinine (µmol/L)	88.0 [71.8–92.3]	77.5 [67.5–83.5]	0.027	88.8 ± 30.3	82.3 ± 35.8	0.084	0.002
Urates (µmol/L)	419 ± 71 †	355 ± 85	0.016	408 [363–440]	399 [360–432]	0.375	0.697
Sodium (mmol/L)	138 ± 3	140 ± 3	0.257	139 ± 2	138 ± 3	0.193	0.733
Potassium (mmol/L)	4.4 ± 0.3	4.4 ± 0.4	0.882	4.3 ± 0.4	4.2 ± 0.5	0.621	0.967
Calcium (mmol/l)	2.7 ± 0.7	2.7 ± 0.8	0.410	2.4 ± 0.1	2.4 ± 1.0	0.198	0.008
Iron (µmol/L)	14.0 ± 4.0	12.6 ± 3.0	0.380	12.3 ± 4.9	14.6 ± 3.4	0.307	0.993
Transferrin (g/L)	2.5 [2.3–2.8]	2.5 [2.4–2.7]	0.910	2.3 ± 0.3	2.2 ± 0.3	0.189	0.951
Glucose (mmol/L)	6.7 ± 1.6	6.2 ± 1.0	0.125	6.8 ± 1.0	6.6 ± 1.9	0.857	0.339
hsCRP (mg/L)	5.4 [1.1–12.1]	1.8 [1.0–3.9]	0.432	13.7 [1.5–30.9]	3.7 [1.8–12.3]	0.055	<0.001
**Chronic CAD patients (Ch-CAD)**							
Erythrocytes (×10^−12^/L)	4.8 ± 0.4	4.7 ± 0.5	0.164	4.6 ± 0.5	4.4 ± 0.4	0.054	0.162
Hemoglobin (g/L)	146 ± 13	144 ± 15	0.177	142 ± 12	137 ± 13	0.035	0.458
Hematocrit (%)	41.9 ± 3.3	40.9 ± 4.1	0.217	40.9 ± 3.1	39.3 ± 3.2	0.05	0.537
Leukocytes (×10^−9^/L)	8.6 ± 2.9	8.5 ± 1.7	0.928	8.2 ± 2.3	7.5 ± 1.7	0.163	0.913
Thrombocytes (×10^−9^/L)	260 ± 52	244 ± 57	0.193	229 ± 39	214 ± 36	0.054	0.510
vWf	1.3 ± 0.4	1.3 ± 0.5	0.794	1.6 ± 0.7	1.5 ± 0.5	0.251	0.629
Urea (mmol/L)	6.8 ± 1.8	7.4 ± 3.2	0.455	6.2 [5.2–7.5]	6.5 [5.4–7.7]	0.426	0.733
Creatinine (µmol/L)	73.5 [63.8–92.8]	75.0 [55.5–81.8]	0.027	95 ± 75	95 ± 77	0.815	0.031
Urates (µmol/L)	330 ± 130	310 ± 104	0.161	340 ± 81	329 ± 79	0.403	0.606
Sodium (mmol/L)	138 ± 3	139 ± 2	0.876	139 ± 2	139 ± 2	0.297	0.106
Potassium (mmol/L)	4.4 ± 0.2	4.6 ± 0.4	0.415	4.4 ± 0.3	4.2 ± 0.2	0.127	0.343
Calcium (mmol/L)	2.47 [2.41–2.56]	2.46 [2.39–2.52]	0.496	2.46 [2.34–2.54]	2.44 [2.37–2.49]	0.275	0.935
Iron (µmol/L)	15.7 ± 5.1	14.1 ± 4.0	0.309	14.9 ± 2.8	14.5 ± 2.9	0.739	0.568
Transferrin (g/L)	2.5 [2.4–2.8]	2.7 [2.3–2.8]	0.820	2.5 ± 0.4	2.4 ± 0.4	0.295	0.055
Glucose (mmol/L)	6.7 [5.8 - 8.4]	6.9 [5.6–8.9]	0.846	6.5 ± 1.5	6.6 ± 1.4	0.812	0.167
hsCRP (mg/L)	3.3 [1.0–5.5]	2.8 [0.5–7.9]	0.922	1.7 ± 1.1	1.6 ± 1.0	0.779	0.577

Data are presented as the mean ± standard deviation (SD) (normally distributed data) or as the median and interquartile range (non-normally distributed data). Total study population: control N = 20 (women N = 3, men N = 17) and n-3 PUFAs N = 20 (women N = 6, men N = 14). Acute CAD patients (Ac-CAD): control N = 10 (women N = 1, men N = 9) and n-3 PUFAs N = 10 (women N = 1, men N = 9). Chronic CAD patients (Ch-CAD): control N = 10 (women N = 2, men N = 8) and n-3 PUFAs N = 10 (women N = 5, men N = 5). n-3 PUFAs—n-3 polyunsaturated fatty acids; hsCRP—high sensitivity C-reactive protein; vWf—von Willebrand factor (VWF). *^a^* Analysis of difference between before and after within the group (paired *t*-test or Wilcoxon signed-rank test). *^b^* Analysis of covariance (ANCOVA) model included the baseline value as the covariate.

**Table 4:** Effect of regular (control group) and n-3 PUFA-enriched hen egg (n-3 PUFAs group) consumption on serum lipid profile in the total population (CAD) and in the two subgroups (Ac-CAD patients and Ch-CAD patients).

Parameter	Control			n-3 PUFAs			Between-Group Effect (After) Adjusted for Baseline
	Before	After	*p* Value *^a^*	Before	After	*p* Value *^a^*	*p* Value *^b^*
**Total study population (CAD)**							
Cholesterol (mmol/L)	4.2 ± 1.1	3.8 ± 0.9	0.118	4.6 ± 0.8	4.1 ± 0.8 †	0.022	0.613
Triglycerides (mmol/L)	1.8 ± 0.9	1.5 ± 0.6	0.045	1.6 [1.1–2.5]	1.4 [1.0–1.8]	0.349	0.455
HDL cholesterol (mmol/L)	1.0 ± 0.3	1.0 ± 0.3	0.786	1.1 [1.0–1.4]	1.1 [0.8–1.4]	0.275	0.613
LDL cholesterol (mmol/L)	2.6 ± 0.9	2.3 ± 0.6	0.120	2.8 ± 0.7	2.4 ± 0.6	0.011	0.335
**Acute CAD patients (Ac-CAD)**							
Cholesterol (mmol/L)	4.6 ± 1.1	3.7 ± 0.6	0.037	4.9 ± 0.9	3.9 ± 0.6	<0.001	0.169
Triglycerides (mmol/L)	2.1 ± 0.9	1.5 ± 0.5	0.048	2.0 [1.4–2.7]	1.6 [1.1–2.5]	0.557	0.306
HDL cholesterol (mmol/L)	0.9 ± 0.2	0.9 ± 0.2	0.875	0.9 ± 0.1	0.9 ± 0.2	0.491	0.440
LDL cholesterol (mmol/L)	2.8 [2.4–3.7]	2.2 [2.0–2.6]	0.129	3.1 ± 0.8	2.3 ± 0.4	0.002	0.138
**Chronic CAD patients (Ch-CAD)**							
Cholesterol (mmol/L)	3.9 ± 1.1	4.0 ± 1.1	0.471	4.2 ± 0.6	4.4 ± 0.9	0.415	0.217
Triglycerides (mmol/L)	1.6 ± 0.8	1.4 ± 0.7	0.510	1.3 [0.9–2.0]	1.2 [1.0–1.7]	1.000	0.428
HDL cholesterol (mmol/L)	1.1 ± 0.3	1.1 ± 0.3	0.688	1.3 [1.1–1.4]	1.3 [1.1–1.6]	0.240	0.557
LDL cholesterol (mmol/L)	2.3 ± 0.7	2.4 ± 0.7	0.521	2.5 ± 0.6	2.4 ± 0.7	0.641	0.821

Data are presented as the mean ± standard deviation (SD) (normally distributed data) or as the median and interquartile range (non-normally distributed data). Total study population: control N = 20 (women N = 3, men N = 17) and n-3 PUFAs N = 20 (women N = 6, men N = 14). Acute CAD patients (Ac-CAD): control N=10 (women N = 1, men N = 9) and n-3 PUFAs N = 10 (women N = 1, men N = 9). Chronic CAD patients (Ch-CAD): control N = 10 (women N = 2, men N = 8) and n-3 PUFAs N = 10 (women N = 5, men N = 5). n-3 PUFAs—n-3 polyunsaturated fatty acids; HDL—high-density lipoprotein; LDL—low-density lipoprotein. *^a^* Analysis of difference between before and after within the group (paired *t*-test or Wilcoxon signed-rank test). *^b^* Analysis of covariance (ANCOVA) model included the baseline value as the covariate.

**Table 5:** Effect of regular (control group) and n-3 PUFA-enriched hen eggs (n-3 PUFAs group) consumption on serum free fatty acid profile in cardiovascular (CAD) patients.

Parameter		Control Group			n-3 PUFAs Group			Between-Group Effect (After) Adjusted for Baseline
		Before	After	*p* Value *^b^*	Before	After	*p* Value *^b^*	*p* Value *^c^*
**SFA (μmol/L)**								
	C14:0 Myristic acid	31.2 ± 10.5	26.9	NA	29.0 ± 3.3	28.0 ± 1.0	0.982	NA
	C16:0 Palmitic Acid	369 ± 156	250 ± 43	0.268	348 ± 49	262 ± 59	0.003	0.058
	C18:0 Stearic acid	111 ± 52	75 ± 7	0.281	95 ± 11	91 ± 13	0.421	0.087
**PUFA (μmol/L)**								
n-7	C16:1[cis-9] Palmitoleic acid	39.4 ± 19.2	26.1 ± 11.1	0.305	48.7 ± 13.4	33.4 ± 11.8	<0.001	0.173
n-9	C18:1[cis-9] Oleic acid	381 ± 279	225 ± 44	0.374	250 ± 75	194 ± 55	0.012	0.045
n-6	C18:2[cis-9,12] Linoleic acid	416 ± 252	297 ± 30	0.388	371 ± 83	309 ± 91	0.191	0.251
	C18:3[cis-6,9,12] gamma-Linolenic acid	11.6 ± 1.9	11.6 ± 2.5	0.944	13.4 ± 1.4	11.7 ± 0.2	0.195	0.417
	C20:3[cis-8,11,14] Dihomo-gamma-linolenic acid	24.1 ± 10.1	15.0 ± 1.6	0.165	29.9 ± 7.8	21.2 ± 3.3	0.034	0.059
	C20:4[cis-5,8,11,14] Arachidonic acid	138 ± 17	123 ± 11	0.333	168 ± 44	118 ± 17	0.050	0.066
n-3	C18:3[cis-9,12,15] alpha-Linolenic acid	35.9	<LOQ	NA	<LOQ	15.3	NA	NA
	C20:4[cis-5,8,11,14] Eicosa-5,8,11,14,17-pentaenoic acid	<LOQ	<LOQ	NA	11.7 ± 3.0	14.4 ± 3.0	0.002	NA
	C22:6[cis-4,7,10,13,16,19] cis-4,7,10,13,16,19-Docosahexaenoic acid	24.9 ± 16.2	19.9 ± 3.9	0.537	22.2 ± 5.3	29.7 ± 9.6	0.148	0.324
	**n6/n3 PUFAs**	5.8	6.3		8.3	5.5		

Data are presented as the mean ± standard deviation (SD) (normally distributed data) or as the median and interquartile range (non-normally distributed data). Analysis of Ac-CAD randomly selected 8 samples: control N = 4 and n-3 PUFAs N = 4. <LOQ—below limit of quantification: C12:0 lauric acid, C15:0 pentadecylic acid, C17:0 margaric acid, C20:0 arachidic acid, C22:0 behenic acid, C23:0 tricosanoic acid, C24:0 lignoceric acid, C14:1[cis-9] myristoleic acid, C17:1[cis-10] cis-10-heptadecenoic acid, C20:1[cis-11] 11-eicosenoic acid, C24:1[cis-15] nervonic acid, C21:2[cis-11,14] eicosadienoic acid. N/F—not found: C4:0 butyric acid, C6:0 caproic acid, C8:0 caprylic acid, C10:0 capric acid, C11:0 undecylic acid, C13:0 tridecylic acid, C21:0 heneicosanoic acid, C15:1[cis-10] Cis-10-pentadecenoic acid, C18:1[trans-9] elaidic acid, C22:1[cis-13] erucic acid, C18:2[trans-9,12] linoelaidic acid, C22:2[cis-13,16] 13,16-docosadienoic acid, C20:3[cis-11,14,17] 11,14,17-eicosatrienoic acid. *^a^* Analysis of difference between before and after within the group (paired *t*-test or Wilcoxon signed-rank test). *^b^* Analysis of covariance (ANCOVA) model included the baseline value as the covariate. *^c^* *p* < 0.05 difference in baseline value between Control and n-3 PUFAs (Student’s *t*-test).

**Table 6:** Effect of regular (control group) and n-3 PUFA-enriched hen eggs (n-3 PUFAs group) consumption on serum pro- and anti-inflammatory cytokines and chemokines protein concentration in the total population (CV) and in the two subgroups (Ac-CV patients and Ch-CV patients).

Parameter (pg/mL)	Control			n-3 PUFAs			Between-Group Effect (After) Adjusted for Baseline
	Before	After	*p* Value *^a^*	Before	After	*p* Value *^a^*	*p* Value *^b^*
**Total study population (CAD)**							
IL-1a	0.35 [0.30–0.40]	0.35 [0.30–0.40]	0.025	0.35 [0.30–0.40]	0.30 [0.20–0.45]	0.047	0.411
TNF-α	2.1 [1.9–2.6]	1.9 [1.2–3.0]	0.804	1.76 ± 0.74	1.85 ± 0.85	0.729	0.241
IL-6	2.5 [2.0–3.0]	2.5 [2.5–3.0]	1.000	3.0 [2.5–3.0]	2.5 [2.5–3.0]	0.188	0.829
IL-10	1.9 [1.2–2.5]	1.9 [1.2–3.0]	0.638	1.68 ± 1.13	2.11 ± 1.48	0.183	0.367
MCP-1	0.29 [0.06–0.53]	0.41 [0–0.88]	0.174	0.29 [0–0.76]	0.41 [0–1.46]	0.399	0.374
**Acute CAD patients (Ac-CAD)**							
IL-1a	0.35 ± 0.07	0.38 ± 0.07	0.111	0.41 ± 0.10	0.28 ± 0.22	0.122	0.966
TNF-α	1.80 ± 1.00	1.51 ± 1.07	0.490	1.98 ± 0.86	1.69 ± 1.07	0.544	0.700
IL-6	2.48 ± 1.09	2.57 ± 0.59	0.835	3.00 [2.75–4.00]	3.00 [2.50–3.00]	0.047	0.132
IL-10	5.20 ± 11.20	5.59 ± 11.10	0.332	2.09 ± 1.30	2.28 ± 1.36	0.543	0.576
MCP-1	0.33 ± 0.36	0.57 ± 0.44	0.285	0.60 ± 0.81	0.73 ± 1.13	0.585	0.052
**Chronic CAD patients (Ch-CAD)**							
IL-1a	0.37 ± 0.10	0.41 ± 0.13	0.111	0.33 ± 0.13	0.29 ± 0.13	0.121	0.291
TNF-α	2.08 ± 1.05	2.59 ± 2.10	0.476	1.56 ± 0.60	2.00 ± 0.61	0.176	0.418
IL-6	2.47 ± 1.04	2.72 ± 1.40	0.727	2.75 [0.90–3.00]	2.50 [1.88–2.50]	0.844	0.707
IL-10	1.9 [0.6–2.2]	1.9 [0.0–2.5]	1.000	1.31 ± 0.87	1.97 ± 1.65	0.256	0.291
MCP-1	0.18 [0.06–0.82]	0.18 [0.09–0.559]	0.547	0.43 ± 0.52	0.92 ± 0.85	0.083	0.298

Data are presented as the mean ± standard deviation (SD) (normally distributed data) or as the median and interquartile range (non-normally distributed data). Total study population: control N = 20 (women N = 3, men N = 17) and n-3 PUFAs N = 20 (women N = 6, men N = 14). Acute CAD patients (Ac-CAD): control N = 10 (women N = 1, men N = 9) and n-3 PUFAs N = 10 (women N = 1, men N = 9). Chronic CAD patients (Ch-CAD): control N = 10 (women N = 2, men N = 8) and n-3 PUFAs N = 10 (women N = 5, men N = 5). n-3 PUFAs—n-3 polyunsaturated fatty acids; IL-1a—interleukin 1a; TNF-α—tumor necrosis factor alpha; IL-6—interleukin 6; IL-10—interleukin 10; MCP-1—monocyte chemoattractant protein. *^a^* Analysis of difference between before and after within the group (paired *t*-test or Wilcoxon signed-rank test). *^b^* Analysis of covariance (ANCOVA) model included the baseline value as the covariate.

**Table 7:** Effect of regular (control group) and n-3 PUFA-enriched hen eggs (n-3 PUFAs group) consumption on oxidative stress level (TBARS), antioxidant capacity (FRAP), and antioxidant enzyme (GPx) serum activity in the total population (CAD) and in the two subgroups (Ac-CAD patients and Ch-CAD patients).

Parameter (pg/mL)	Control			n-3 PUFAs			Between-Group Effect (After) Adjusted for Baseline
	Before	After	*p* Value *^a^*	Before	After	*p* Value *^a^*	*p* Value *^b^*
**Total study population(CAD)**							
TBARS (uM MDA)	0.370 [0.215–3.035]	0.425 [0.288–3.464]	0.353	0.334 [0.279–0.790]	0.352 [0.297–0.626]	0.890	0.191
FRAP (mM/L Trolox)	0.548 [0.446–0.683]	0.530 [0.401–0.634]	0.071	0.570 ± 0.127	0.535 ± 0.168	0.365	0.682
GPx (U/mg protein)	0.022 ± 0.011	0.007 ± 0.006	0.002	0.022 ± 0.011	0.008 ± 0.005	<0.001	0.724
**Acute CAD patients (Ac-CAD)**							
TBARS (uM MDA)	2.603 ± 2.171	2.558 ± 1.609	0.963	0.790 [0.370–2.615]	0.626 [0.370–3.062]	1.000	0.167
FRAP (mM/L Trolox)	0.628 ± 0.103	0.554 ± 0.083	0.034	0.608 ± 0.135	0.594 ± 0.157	0.842	0.366
GPx (U/mg protein)	0.029 ± 0.004	0.006 ± 0.003	<0.001	0.029 [0.028–0.032]	0.006 [0.004–0.012]	0.016	0.351
**Chronic CAD patients (Ch-CAD)**							
TBARS (uM MDA)	0.222 ± 0.128	0.268 ± 0.130	0.436	0.291 ± 0.118	0.277 ± 0.091	0.399	0.280
FRAP (mM/L Trolox)	0.465 ± 0.139	0.490 ± 0.171	0.727	0.536 ± 0.116	0.482 ± 0.166	0.185	0.258
GPx (U/mg protein)	0.008 ± 0.004	0.009 ± 0.003	0.858	0.014 ± 0.004 ^c^	0.009 ± 0.006	0.030	0.561

Data are presented as the mean ± standard deviation (SD) (normally distributed data) or as the median and interquartile range (non-normally distributed data). Total study population: control N = 20 (women N = 3, men N = 17) and n-3 PUFAs N = 20 (women N = 6, men N = 14). Acute CAD patients (Ac-CAD): control N=10 (women N = 1, men N = 9) and n-3 PUFAs N = 10 (women N = 1, men N = 9). Chronic CAD patients (Ch-CAD): control N = 10 (women N = 2, men N = 8) and n-3 PUFAs N = 10 (women N = 5, men N = 5). n-3 PUFAs—n-3 polyunsaturated fatty acids; CAD—coronary artery disease; TBARS—thiobarbituric acid-reactive substances; FRAP—ferric-reducing ability of plasma; GPx—glutathione peroxidase. *^a^* Analysis of difference between before and after within the group (paired *t*-test or Wilcoxon signed-rank test). *^b^* Analysis of covariance (ANCOVA) model included the baseline value as the covariate. *^c^* *p* < 0.05 difference in baseline value between Control and n-3 PUFAs (student *t*-test).

## Data Availability

The data presented in this study are available on request from the corresponding author.

## Supplementary Materials

The following are available online at https://www.mdpi.com/article/10.3390/biology10080774/s1, Laboratory reference range for measured biochemical parameters, Table S1; Body composition and body fluid status responses to regular (control group) and n-3 PUFA-enriched hen egg (n-3 PUFAs group) consumption in the total study population (CAD) and in the two subgroups (Ac-CAD patients and Ch-CAD patients), Table S2: Body composition and body fluid status—measurement and results.
