# Supplementary material for: Effects of n-3 Polyunsaturated Fatty Acid-Enriched Hen Egg Consumption on the Inflammatory Biomarkers and Microvascular Function in Patients with Acute and Chronic Coronary Syndrome—A Randomized Study
Source: Biology (Basel). 2021 Aug 14;10(8):774. doi: 10.3390/biology10080774 (PMC8389665; doi:10.3390/biology10080774)
Supplement: Supplementary file 1 [file biology-10-00774-s001.zip › biology-1251708- supplementary revised.pdf]

## Supplementary Material

### *Body Composition and Body Fluid Status - Measurement*

Body composition and body fluid status were measured using a four-terminal portable impedance analyzer (Maltron Bioscan 920-II, Maltron International Ltd., Rayleigh, Essex, UK). Empirically derived formulas (the original manufacturer's software) were used to calculate the estimated Fat Free Mass% (FFM%), Fat% (Fat Mass%), Total Body Water% (TBW%), Extracellular Water% (ECW%), Intracellular Water% (ICW%), Plasma Fluid (PF), Interstitial Fluid (IF) and Body Density (kg/L).

### *Body composition and body fluid status - Results*

*Total study (CAD) population and Ch-CAD subgroup.* There was no significant difference in body composition and body fluid status after consumption of three n-3 PUFAs or regular hen eggs compared to initial measurements within the n-3 PUFAs or Control group.

*Ac-CAD subgroup.* There was no significant difference in body composition and body fluid status after consumption of three n-3 PUFAs enriched hen eggs compared to initial measurements within the n-3 PUFAs group. Consumption of regular hen eggs significantly increased fat free mass, total body water, plasma fluid and interstitial fluid, and decreased fat mass, while other measured parameters remained unchanged following regular eggs consumption compared to initial measurement in Control. All results are presented in Table S1, in paper supplement.

**Table S1. Body composition and body fluid status responses to regular (Control group) and n-3 PUFAs enriched hen eggs (n-3 PUFAs group) consumption in total study (CAD) population and in two subgroups (Ac-CAD patients and Ch-CAD patients)**

| Parameter | **Control** | | | **n-3 PUFAs** | | | Between group effect (after) adjusted for baseline |
|---|---|---|---|---|---|---|---|
| | **before** | **after** | *P value*[a] | **before** | **after** | *P value*[a] | *P value*[b] |
| **Total study (CV) population** | | | | | | | |
| Fat Free Mass (%) | 66.4 [60.5 - 71.3] | 66.3 [61.1 - 71.3] | *0.182* | 65.5 ± 10.3 | 65.3 ± 10.9 | *0.996* | *0.190* |
| Fat (%) | 33.6 [28.7 - 39.5] | 33.7 [28.7 - 38.9] | *0.182* | 34.5 ± 10.3 | 34.7 ± 10.9 | *0.996* | *0.196* |
| Total Body Water (%) | 48.9 [46.1 - 50.8] | 48.7 [46.6 - 51.2] | *0.312* | 50.6 ± 12.2 | 50.5 ± 12.9 | *0.855* | *0.173* |
| Extracellular Water (%) | 44.7 ± 2.9 | 44.9 ± 2.9 | *0.964* | 44.3 [42.6 - 46.2] | 44.1 [43.6 - 45.5] | *0.927* | *0.056* |
| Intracellular Water (%) | 55.3 ± 2.9 | 55.1 ± 2.9 | *0.964* | 55.7 [53.8 - 57.4] | 55.9 [54.5 - 56.4] | *0.927* | *0.055* |
| Plasma Fluid (L) | 4.25 ± 1.04 | 4.75 ± 2.30 | *0.312* | 4.09 ± 1.21 | 4.04 ± 0.94 | *0.741* | *0.824* |
| Interstitial Fluid (L) | 14.87 ± 3.63 | 15.04 ± 3.89 | *0.626* | 14.29 ± 4.24 | 14.16 ± 3.27 | *0.778* | ***0.024*** |
| Body Density (kg/L) | 1.024 [1.011 - 1.030] | 1.023 [1.012 - 1.030] | *0.463* | 1.030 [1.005 - 1.040] | 1.030 [1.013 - 1.039] | *0.846* | *0.263* |
| **Acute CV patients (Ac-CV)** | | | | | | | |
| Fat Free Mass (%) | 66.5 [60.3 - 71.6] | 68.8 [60.9 - 73.5] | ***0.044*** | 70.6 ± 5.9 | 69.7 ± 7.8 | *0.359* | *0.418* |

| | | | | | | | |
|---|---|---|---|---|---|---|---|
| Fat (%) | 33.5 [28.5 - 39.7] | 31.2 [26.6 - 39.3] | ***0.044*** | 29.4 ± 5.9 | 30.3 ± 7.8 | *0.359* | *0.451* |
| Total Body Water (%) | 48.2 [45.1 - 51.7] | 49.0 [45.7 - 56.4] | ***0.039*** | 57.4 ± 12.9 | 56.5 ± 14.5 | *0.317* | *0.943* |
| Extracellular Water (%) | 44.4 ± 3.9 | 44.9 ± 3.7 | *0.402* | 45.4 ± 2.4 | 43.9 ± 1.6 | *0.164* | *0.230* |
| Intracellular Water (%) | 55.6 ± 3.9 | 55.1 ± 3.7 | *0.402* | 54.6 [52.2 - 56.4] | 56.1 [54.6 - 56.5] | *0.164* | ***0.042*** |
| Plasma Fluid (L) | 4.02 [3.29 - 4.75] | 4.42 [3.41 - 6.25] | ***0.045*** | 4.80 ± 1.27 | 4.48 ± 1.01 | *0.063* | *0.937* |
| Interstitial Fluid (L) | 14.07 [11.49 - 16.60] | 14.77 [11.94 - 18.83] | ***0.039*** | 16.80 ± 4.47 | 15.71 ± 3.51 | *0.075* | *0.155* |
| Body Density (kg/L) | 1.025 [1.012 - 1.031] | 1.027 [1.008 - 1.039 ] | *0.297* | 1.034 ± 0.012 | 1.032 ± 0.017 | *0.280* | *0.173* |
| **Chronic CV patients (Ch-CV)** | | | | | | | |
| Fat Free Mass (%) | 66.4 [60.3 - 71.2] | 66.0 [61.0 - 69.8] | *1.000* | 60.3 ± 11.3 | 61.1 ± 12.1 | *0.494* | *0.088* |
| Fat (%) | 33.6 [28.8 - 39.7] | 34.0 [30.2 - 39.0] | *1.000* | 39.7 ± 11.3 | 38.9 ± 12.1 | *0.494* | *0.087* |
| Total Body Water (%) | 50.3 [46.5 - 51.3] | 48.7 [47.1 - 50.4] | *0.625* | 43.8 ± 6.8 | 44.5 ± 8.0 | *0.575* | *0.063* |
| Extracellular Water (%) | 45.1 ± 1.5 | 44.7 ± 2.2 | *0.432* | 43.3 [41.8 - 45.0] | 45.0 [43.4 - 46.0] | *0.232* | *0.207* |
| Intracellular Water (%) | 54.9 ± 1.5 | 55.3 ± 2.2 | *0.432* | 56.7 [55.0 - 58.2] | 54.0 ± 5.2 | *0.232* | *0.207* |
| Plasma Fluid (L) | 4.15 ± 0.46 | 3.99 ± 0.61 | *0.169* | 3.37 ± 0.57 | 3.60 ± 0.64 | *0.207* | *0.294* |
| Interstitial Fluid (L) | 14.52 ± 1.62 | 13.97 ± 2.13 | *0.168* | 11.78 ± 1.99 | 12.61 ± 2.23 | *0.206* | *0.296* |
| Body Density (kg/L) | 1.024 [1.011 - 1.031] | 1.023 [1.012 - 1.030] | *0.813* | 1.016 [0.988 - 1.030] | 1.022 [0.980 - 1.030] | *0.625* | *0.073* |

Data are presented as mean ± standard deviation (SD) (normally distributed data) or as median and interquartile range (not normally distributed data)

Total study population: Control N=20 (women N=3, men N=17) and n-3 PUFAs N=20 (women N=6, men N=14)

Acute CAD patients (Ac-CAD): Control N=10 (women N=1, men N=9) and n-3 PUFAs N=10 (women N=1, men N=9)

Chronic CAD patients (Ch-CAD) : Control N=10 (women N=2, men N=8) and n-3 PUFAs N=10 (women N=5, men N=5)

n-3 PUFAs- n-3 polyunsaturated fatty acids; CAD- coronary artery disease.

[a] analysis of difference between before and after within the group (paired t-test or Wilcoxon Signed Rank Test)

[b] analysis of covariance (ANCOVA) model included baseline value as covariate

Laboratory reference range for measured biochemical parameters is listed in Table S2.

**Table S2. Laboratory reference range for measured biochemical parameters from venous blood samples**

| Parameter | lab reference range |
|---|---|
| erythrocytes (x10E12/L) | 4.34 - 5.72 |
| hemoglobin (g/L) | 138 - 175 |
| hematocrit (%) | 41.5 - 53 |

| | |
|---|---|
| leukocytes (x10E9/L) | 3.4 - 9.7 |
| thrombocytes (x10E9/L) | 158 - 424 |
| vWf | 0.50 - 1.50 |
| urea (mmol/L) | 2.8 - 8.3 |
| creatinine (μmol/l) | 64 - 104 |
| urates (μmol/l) | 182 - 403 |
| sodium (mmol/l) | 137 - 146 |
| potassium (mmol/l) | 3.9 - 5.1 |
| calcium (mmol/l) | 2.14 - 2.53 |
| iron (μmol/l) | 11.0 - 32.0 |
| transferrin (g/l) | 2.0 - 3.6 |
| glucose (mmol/L) | 4.4 - 6.4 |
| hsCRP (mg/L) | < 5 |
| cholesterol (mmol/L) | < 5 |
| triglycerides (mmol/L) | < 1.7 |
| HDL cholesterol (mmol/L) | > 1 |
| LDL cholesterol (mmol/L) | < 2.62 |

hsCRP- high sensitivity C reactive protein
